# Norwegian PUQE (Pregnancy-Unique Quantification of Emesis and Nausea) Identifies Patients with Hyperemesis Gravidarum and Poor Nutritional Intake: A Prospective Cohort Validation Study

**DOI:** 10.1371/journal.pone.0119962

**Published:** 2015-04-01

**Authors:** Elisabeth Birkeland, Guro Stokke, Randi J. Tangvik, Erik A. Torkildsen, Jane Boateng, Anne L. Wollen, Susanne Albrechtsen, Hans Flaatten, Jone Trovik

**Affiliations:** 1 Dpt. Clinical Science, University of Bergen, Bergen, Norway; 2 Dpt. Obstetrics and Gynecology, Haukeland University Hospital, Bergen, Norway; 3 Dpt. Research and Development, Haukeland University Hospital, Bergen, Norway; 4 Dpt. Obstetrics and Gynecology, Stavanger University Hospital, Stavanger, Norway; 5 Dpt. Obstetrics and Gynecology, Helse-Foerde, Foerde, Norway; 6 Dpt. Bergen, Volvat Medical Centre, Bergen, Norway; 7 Dpt. Anesthesia and Intensive Care, Haukeland University Hospital, Bergen, Norway; 8 Dpt. Clinical Medicine, University of Bergen, Bergen, Norway; Oslo University Hospital, Ullevål, NORWAY

## Abstract

**Objective:**

The English questionnaire Pregnancy-Unique Quantification of Emesis and nausea (PUQE) identifies women with severe Hyperemesis Gravidarum. Our aim was to investigate whether scores from the translated Norwegian version; SUKK (SvangerskapsUtløst Kvalme Kvantifisering) was associated with severity of hyperemesis and nutritional intake.

**Design:**

A prospective cohort validation study.

**Setting:**

Hospital cohort of Hyperemesis Gravidarum (HG) patients from western Norway and healthy pregnant women from Bergen, Norway.

**Sample:**

38 women hospitalized due to HG and 31 healthy pregnant controls attending routine antenatal check-up at health centers.

**Methods:**

Data were collected May 2013-January 2014. The study participants answered the Norwegian PUQE-questionnaire (scores ranging from 3 to15) and registered prospectively 24-hours nutritional intake by a food list form.

**Main outcome measures:**

Differences of PUQE-scores, QOL-score and nutritional intake between hyperemesis patients and controls.

**Results:**

Hyperemesis patients had shorter gestational age compared to controls (median 9.7 weeks; 95% CI 8.6-10.6 versus 11.9; 95% CI 10.1-12.9, *p*=0.004), and larger weight-change from pre-pregnant weight (loss of median 3 kg; 95% CI 3-4 versus gain of 2 kg; 95% CI 0.5-2, *p*<0.001) otherwise groups were similar regarding pre-pregnant BMI, age, gravidity, and inclusion weight. Compared to controls, hyperemesis patients had significant higher PUQE-score (median 13; 95% CI 11-14 vs. 7; 95% CI 4-8), lower QOL (median score 3; 95% CI 2-4 vs. 6; 95% CI 4.5-8) and lower nutritional intake (energy intake median 990 kcal/24 hours; 95% CI 709-1233 vs. 1652; 95% CI 1558-1880 all *p*<0.001). PUQE-score was inversely correlated to nutritional intake (-0.5, *p*<0.001). At discharge PUQE-score had fallen to median 6 (95% CI 5-8) and QOL score risen to 7 (95% CI 6-8) in the HG group, (both *p*<0.001 compared to admission values).

**Conclusion:**

PUQE-scoring has been validated as a robust indicator of severe hyperemesis gravidarum and insufficient nutritional intake in a Norwegian setting.

## Introduction

Nausea and vomiting occur in up to 80% of all pregnancies [1]. The condition, although associated with significant reduced quality of life [2], is mostly self-limiting. About 0.3–1.5% of pregnant women develop the more serious condition Hyperemesis Gravidarum (HG) [3]. HG as defined by Fairweather is vomiting occurring in pregnancy before the twentieth week of gestation, of such severity as to require the patient’s admission to hospital, and being unassociated with other coincidental conditions causing vomiting [4]. In the International Classification of Diseases (ICD-10) the diagnosis O21.1 is Hyperemesis with metabolic disturbance occurring before 22^nd^ weeks of pregnancy. The etiology of HG is unknown.

Persistent low food intake and/or frequent vomiting can lead to dehydration, metabolic imbalance, nutritional deficiencies and weight loss [3]. Severe maternal weight loss in early pregnancy or insufficient catch-up weight has been linked with unfavorable fetal outcomes such as preterm delivery and growth restriction [5,6].

No single measure can easily define, quantify or evaluate the treatment of hyperemesis, but an English pregnancy specific questionnaire PUQE (Pregnancy-Unique Quantification of Emesis) has been developed in order to assess the severity of emesis (nausea and vomiting) in pregnancy [7]. This questionnaire contains three questions regarding the time-span of nausea, vomiting and retching respectively, as well as one question assessing the global psychological and physical quality of life (QOL). Initially the questionnaire evaluated symptoms during last 12 hours, but it has been modified to encompass 24 hours [8] as well as the whole of first trimester of pregnancy [9]. PUQE-score has been validated to correlate with inability of taking iron supplementation in pregnancy, risk of hospitalization due to HG or severe nausea and vomiting in pregnancy (NVP), increased health care costs due to NVP and reduced well-being/QOL [10]. The PUQE questionnaire has also been used in several studies assessing effect of antiemetic treatments for emesis and hyperemesis [8–12], but studies evaluating PUQE in relation to the woman’s nutritional intake are lacking.

Although PUQE has been translated and used in several languages; Indonesian [13], Turkish [14], Italian [15], French [9] and Spanish [8], no Scandinavian version had yet been developed. This translation and our validation study was undertaken to introduce PUQE as a tool for HG diagnostics and evaluation in Norway.

This study thus aimed to validate in a Norwegian population if “SvangerskapsUtløst Kvalme Kvantifisering” (SUKK), the Norwegian translated version of PUQE-24, significantly differentiates between emesis commonly occurring in pregnancy and severe hyperemesis. Additionally, we wanted to compare the food intake between women with severe NVP/HG and healthy pregnant women and relate this to their PUQE-scores.

## Material and Methods

A prospective cohort validation study was conducted where SUKK, the Norwegian version of the PUQE-24 questionnaire and a nutrition diary were completed by patients hospitalized due to HG and compared to those from a group of healthy pregnant women (controls).

The participating women with hyperemesis were included from Department of Gynecology at three hospitals in Western Norway; Bergen, Foerde and Stavanger. The control group was recruited at health care centers in Bergen. Inclusion period was between May the 1^st^ 2013 and end of January 2014.

The Norwegian Regional Ethical Committee (REK Norway) and the Institutional Boards have approved this study (2013/465). All participants signed consent at inclusion. The study is registered at ClinicalTrials.gov (NCT01836835)[16].

The inclusion criteria for the HG group were women hospitalized due to severe nausea and vomiting in pregnancy with at least two out of three criteria; dehydration, weight loss or electrolyte imbalance/ketonuria. Inclusion criterion for the control group was a healthy on-going pregnancy in low-risk women attending routine antenatal care. Women were excluded if they were unable to understand and read/write Norwegian, suffered from other diseases causing nausea and vomiting or if the gestational length was more than 16 weeks at inclusion. Control women would not be included if they primarily attended the physician to receive treatment for nausea/emesis. The fulfillments of eligibility were verified by the responsible gynecologist at each hospital and by the physicians/health personnel responsible for outpatient pregnancy controls. No age or parity matching was performed.

Forms from included hyperemesis patients were collected by the recruiting physicians while forms from each control woman were mailed directly to the primary investigator.

Demographic data including parity, previous HG pregnancies, date of last menstruation, height and weight before pregnancy and at inclusion were self-reported by the women. The patients’ self-reported weight at inclusion was checked with weight at admission reported in the hospital’s patient file. Gestational age was calculated according to date of last menstruation unless sonography dating had been performed and was stated in the inclusion form.

**Fig 1:**
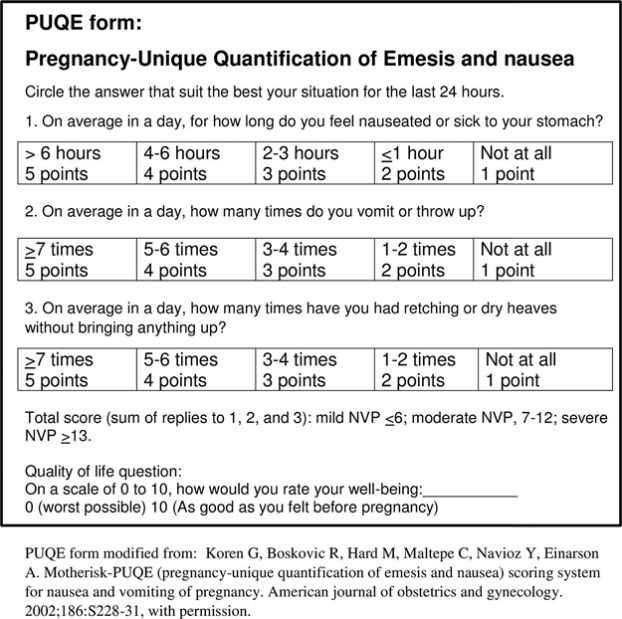
PUQE-24*-questionnaire used in prospective cohort validation study of HG^ versus healthy pregnant women. *Pregnancy-Unique Quantification of Emesis and nausea. ^Hyperemesis Gravidarum.

The PUQE-24 questionnaire, including the quality of life question (Fig. 1), was translated into Norwegian (S1 Fig.) by an authorized translator and independently translated back to English. The back-translated version was approved by the original developer; Dr. Gideon Koren.

The participants reported their background information at inclusion, prospectively completed a Norwegian food and drink intake list during a period of 24 hours and then answered the PUQE-questionnaire. The hyperemesis patients in addition answered the questionnaire at discharge from hospital.

The three PUQE questions each have a rating from 1–5, thus the composite sum (PUQE-score) ranged from 3–15. A score between 3–6 points was defined as mild NVP, 7–12 points as moderate NVP and scores ≥13 points was classified as severe NVP/HG, in line with former studies [9,10].

The QOL question, similar as the one used in the original PUQE validations [10], was a rating scale of the woman’s wellbeing at present with a range between zero (the worst possibly imaginable) and ten (equaled as good as she felt before the start of this pregnancy).

PUQE-scores and QOL-scores were compared between patients and controls and for patients between their scores at admission and discharge.

Food and drink intake during 24 hours was registered using a food list form slightly modified from the Norwegian national recommendation for prevention and treatment of malnutrition [17] (S1 Table), including 38 regular food items and fluids. Dinner, dessert, soup, cakes, desserts and toppings for bread slices were specified as per portion. Thus to perform the nutritional calculations for each of these food categories we constructed a mean nutrient intake out of four different common Norwegian choices.

Calculations of reported nutrient intake were performed by using the nutrient analysis program Dietist XP (version 2012, Kost och Naringsdata, Bromma, Sweden). Dietist XP is based on the Swedish National Food Agency (NFA, Livsmedelsverket) and is routinely used by Norwegian nutritionists.

The women’s total energy intake as well as macronutrients (fat, protein, carbohydrate and fiber) and some micronutrients (vitamin D, vitamin C, Vitamin B_12_, Calcium, Iron, Magnesium and Sodium) were calculated. Values for energy, macro- and micronutrients were computed for each participant and compared between the HG and control group as well as between the three-category PUQE-scores (mild, moderate, severe NVP/HG). The participants’ food intake were additionally compared with the Norwegian recommendations of nutrient intake for pregnant women [18].

Based on results from a Canadian study [10] with a mean PUQE-score of 11 ± 3 in the HG group and 9 ± 2.2 in the control group, aiming for alpha = 5% (two sided) and a power of 80%, a sample size of 28 in each group was calculated as sufficient. Similarly using energy intake measured in a South-African study of hyperemesis patients [19] a sample size of 28 was estimated to yield a 100% power to detect significant differences in nutritional intake between patients and normal pregnant women.

All data were recorded anonymously. Statistical analyses were performed using IBM SPSS (Statistical Package for the Social Sciences) version 21 (IBM, Armonk, NY). Statistical significance was set at *p* < 0.05. All tests were two-sided. Chi-square test was used to compare categorical variables. Due to small, not normally distributed data samples we mainly used non-parametric tests to compare the linear variables; Mann-Whitney U test for two independent groups, Kruskal-Wallis test if three or more groups were compared. For related groups (comparing values at admission to discharge) Wilcoxon Signed-Rank Test was performed. This measurement would be used for assessing responsiveness of the PUQE questionnaire. Linear regression was performed to explore the different relation of gestational length and PUQE-score for the two diagnosis groups. Spearman’s rank correlation was used assessing correlation between PUQE-score and QOL-score. The Cronbach’s alpha was used to report the internal consistency of the PUQE-score. Construct validity was assessed by measuring association between PUQE-scores and QOL-scores, nutritional intake and weigh-change using Kruskal-Wallis test. The hypothesis was that high PUQE-sores correlate with low QOL-scores, low nutritional intake and weight loss rather than weight gain (measured from preconception to inclusion). Floor and ceiling effects have been evaluated by demonstrating proportions of participants answering in the extreme values (lowest and highest) of the PUQE-score.

Data have been presented in accordance with the STROBE guidelines [20].

## Results

**Fig 2:**
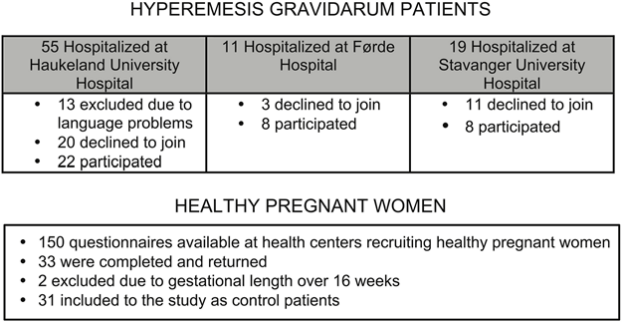
Outline of enrolment in prospective cohort study validating Norwegian PUQE-24* in HG^ patients (n = 38) and healthy pregnant women (n = 31). *Pregnancy-Unique Quantification of Emesis and nausea. ^Hyperemesis Gravidarum.

The flow of participant inclusion in the study is described in Fig. 2. During the inclusion period 85 women were hospitalized due to HG at the three recruiting hospitals. At Haukeland 13 of 55 women were excluded due to language difficulties. Among those invited, a total of 34 declined to participate. Thus 38 included out of total 72 eligible patients yielded a 53% participation rate. 150 questionnaires were administered to those aiming at including healthy pregnant women. 33 women replied, two were excluded due to gestational age above 16 weeks, giving a participation rate of a minimum of 21% as exactly how many women were asked to participate is not known.

Demographic data for the patients and controls are presented in Table 1. Age, gravidity, previously hyperemesis pregnancies or pre-pregnant BMI were not significantly different between the two groups. The HG patients had lost median 3 kg (95% Confidence Interval (CI) 3–4 kg) at admission while the control women had gained median 2 kg (95% CI 0.5–2kg, *p* <0.001 Mann-Whitney U Test). The patients had a significant shorter gestational age at inclusion, median 65 days (95% CI 60–74) as compared to the control group of median 83 days (95% CI 71–90, *p* = 0.004).

**Table 1:** Clinical information from patients hospitalized for HG* and healthy pregnant women.

Variable	HG*patients		Controls		*p*-value
	n = 38		n = 31		Mann-Whitney U Test
	Median	95% CI	Median	95% CI	
Age	28	25–30	30	27–32	0.174
Gravidity (number pregnancies)	2	2–2	2	1–2	0.434
HG in former pregnancies (number)^a^	0.5	0–1	0	0–0	0.189
BMI^b^ before pregnancy (kg/m^2^)	24.9	22.4–26.7	23.3	22.3–25.5	0.286
Weight Inclusion (kg)^c^	65	57–73	67	63–70	0.493
Weight change from pre-conception to inclusion (kg)^c^	-3	-4 – -3	2	0.5–2	<0.001
Height (cm)	167	164–169	167	165–170	0.633
Gestational length (days)	65	60–74	83	71–90	0.004
Days in hospital	2	2–3	-		

*HG: Hyperemesis Gravidarum.

^a^Excluding nullipara: n = 11 in HG* group and n = 13 in controls;

^b^BMI: Body Mass Index;

^c^Weight missing for one healthy control.

In the HG group, QOL answer was missing for one patient at inclusion and three patients at discharge. One lacked PUQE-scores at discharge. Nutrition diary was lacking from one patient. One of the control cases had not registered weight at inclusion, otherwise all data were complete.

**Fig 3:**
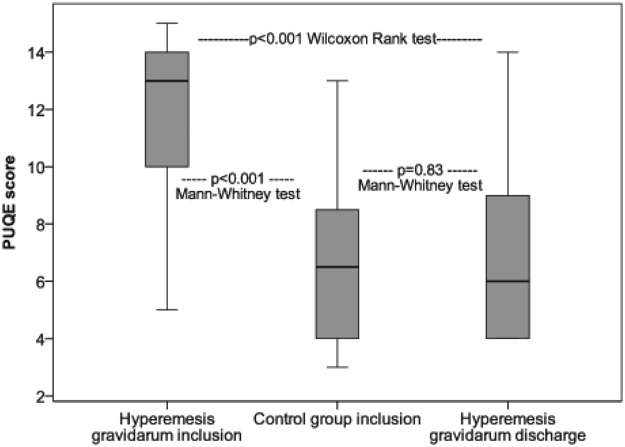
PUQE-24*-score in HG^ patients (n = 38) at admission, healthy pregnant women (n = 31) and HG^ patients (n = 35) at discharge. *Pregnancy-Unique Quantification of Emesis and nausea. ^Hyperemesis Gravidarum.

**Fig 4:**
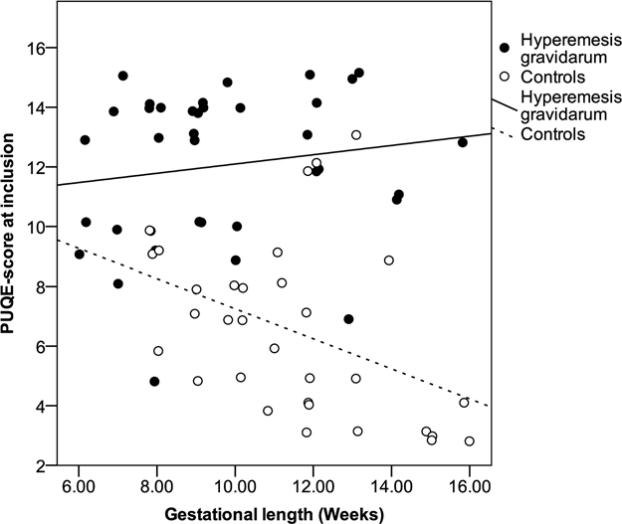
PUQE-24*-score in relation to gestational length for HG^ patients (n = 38) and controls (n = 31). *Pregnancy-Unique Quantification of Emesis and nausea. ^Hyperemesis Gravidarum.

Patients had significantly higher scores in each of the three PUQE questions compared to controls as presented in Table 2. At inclusion the median PUQE-score was 13 in the HG group (95% CI 11–14, range 5–15) while the healthy pregnant women had a median of 7 (95% CI 5–8, range 3–13 *p* <0.001 Mann-Whitney tests), Fig. 3 and S2 Table. As illustrated in Fig. 4, displaying the relation between gestational age and PUQE-score, the two groups had distinct different PUQE-values across the age span investigated. Patients had high PUQE-scores irrespective of gestational length at inclusion while the control group demonstrated decreasing PUQE-scores with higher gestational length. Performing linear regression including the interaction of gestational length and group (hyperemesis group*controls), the overall adjusted model fit was R^2^ = 0.54. The interaction term (gestational age* patients vs. controls) was significant with p = 0.013, demonstrating the different effect of gestational age for the two cohorts included in this study.

**Table 2:** PUQE-24* and Quality of Life scoring in prospective cohort validation study of HG^ and healthy pregnant women.

	Patients		Controls		*p*-value
	n = 38		n = 31		Mann-Whitney U test
	Median	95% CI	Median	95% CI	
Question 1 score (length of nausea)	5	5–5	3	2–4	<0.001
Question 2 score (rate of vomiting)	4	3–4	1	1–1	<0.001
Question 3 score (rate of retching)	4	4–5	2	1–2	<0.001
PUQE-score^a^	13	11–14	7	5–8	<0.001
Quality of life (QOL) score^b^	3	2–4	6	4.5–8	<0.001
PUQE-score severity	Number	%	Number	%	*p*-value
					Chi-square
Mild NVP^c^ (score <7)	1	3	15	48	<0.001
Moderate NVP (score 7–12)	15	40	15	48	
Severe NVP/HG (score 13–15)	22	58	1	3	

*PUQE: Pregnancy-Unique Quantification of Emesis and nausea;

^Hyperemesis Gravidarum;

^a^Sum of Question 1, 2 and 3;

^b^Missing data for one HG patient;

^c^NVP: Nausea and vomiting in pregnancy.

The QOL-score was significantly lower in patients compared to the control group (Table 2, S2 Fig.). The PUQE-score was significantly inverse linearly correlated with QOL-score with r = -0.681 (*p*<0.001 Spearman’s rank correlation). PUQE categories (mild—moderate—severe NVP/HG) were inversely correlated to women’s rating of QOL (Table 3), underlining the burden of hyperemesis; high rate of emesis and nausea leads to significantly reduced quality of life.

**Table 3:** Quality of Life (QOL) score, energy intake and weight change from preconception to inclusion compared to PUQE-24*-score severity.

Variable	Mild NVP^a^		Moderate NVP		Severe NVP/HG		*p-*value
	PUQE-score <7		PUQE-score 7–12		PUQE-score 13–15		Kruskal-Wallis test
	n = 16		n = 29		n = 23		
	Median	95% CI	Median	95% CI	Median	95% CI	
QOL-score^b^	8	7–9.5	4.5	3–5	3	1.5–4	<0.001
Energy intake (kcal/24h)	1796	1558–2031	1408	1171–1605	878	459–1233	<0.001
Weight change from pre-conception to inclusion (kg)	2	2–3	-2.5	-3–0	-3	-5 – -1.2	<0.001

Prospective cohort study of 37 HG^ patients and 31 healthy pregnant women.

*PUQE: Pregnancy-Unique Quantification of Emesis and nausea;

^Hyperemesis Gravidarum;

^a^NVP: Nausea and vomiting of pregnancy;

^b^QOL: Quality Of Life.

At discharge PUQE-score had fallen to median 6 (95% CI 5–8) and QOL-score risen to 7 (both *p*<0.001 compared to values at admission, Wilcoxon Signed-Rank test, Table 4) and were no longer significantly different from those of the control group, all *p*>0.05 Mann-Whitney U Test (S3 Table), see also Fig. 3.

**Table 4:** PUQE*-24-scores from women with Hyperemesis Gravidarum (HG) at admission and discharge from hospital.

Variable	HG hospitalization		HG discharge		*p*-value
	n = 38		n = 37		Wilcoxon Rank test
	Median	95% CI	Median	95% CI	
Question 1 score (length of nausea)	5	5–5	3	2–4	<0.001
Question 2 score (rate of vomiting)	4	3–4	1	1–1	<0.001
Question 3 score (rate of retching)	4	4–5	2	1–2	<0.001
PUQE-score^a^	13	11–14	6	5–8	<0.001
Quality of life (QOL) score ^b^ ^c^	3	2–4	7	6–8	<0.001
PUQE-score severity	Number	%	Number	%	*p*-value
					Chi-square
Mild NVP^d^ (score <7)	1	3	20	54	<0.001
Moderate NVP (score 7–12)	15	40	16	43	
Severe NVP/HG (score 13–15)	22	58	1	3	

*PUQE: Pregnancy Unique Quantification of Emesis and nausea;

^a^Sum of Question 1, 2 and 3;

^b^Data from one participant during hospitalization is missing;

^c^Data from three participants at discharge are missing;

^d^NVP: Nausea and vomiting of pregnancy.

Detailed nutrient parameters are presented in Table 5. HG patients have significantly lower nutritional intake compared to the control group. PUQE-score was inversely correlated to nutritional intake measured as total caloric intake/24hours (r = -0.5, *p*<0.001 Spearman’s rank correlation) also displayed in S3 Fig. Similarly the three PUQE categories had significantly decreasing nutritional intake of each macro- and micronutrients estimated, all *p*≤0.004 as displayed in S4 Table.

**Table 5:** Nutritional intake during 24 hours from patients hospitalized due to Hyperemesis Gravidarum versus healthy pregnant women.

Variable	Patients		Controls		*p*-value
	n = 38		n = 31		Mann-Whitney U Test
	Median	95% CI	Median	95% CI	
Energy intake (kcal^a^)	990	709–1233	1652	1558–1880	<0.001
Protein (g)	28	18–38	63	51–69	<0.001
Fat (g)	37	22–47	66	48–77	<0.001
Carbohydrates (g)	147	99–165	195	167–227	0.001
Vitamin D (μg)	1	1–1	2	1–3	<0.001
Vitamin C (mg)	49	29–65	111	74–154	<0.001
Vitamin B12 (μg)	1	1–1	3	2–3	<0.001
Calcium (mg)	293	181–333	685	545–737	<0.001
Iron (mg)	3	2–4	7	6–9	<0.001
Magnesium (mg)	128	72–157	260	228–301	<0.001
Sodium (mg)	1348	893–1565	1997	1665–2268	<0.001
Fiber (g)	8	6–10	19	114–24	<0.001

^a^kcal: kilocalories.

Norwegian recommendations of a daily caloric intake of 2285 during 1^st^ trimester and 2615 kcal during 2^nd^ trimester [18] were fulfilled by only 1 of 37 patients and 4 of 31 control women. None of 23 participants with PUQE-score ≥13 had a sufficient nutritional intake. The majority of HG patients had an energy intake less than 50% of recommended; 21 patients (57%) as compared to six women (19%) of the control group, *p* = 0.003 Chi-square test.

Analyzing the three different PUQE-questions in relation to the total PUQE-score yields a Cronbach’s alpha of 0.846, documenting a good internal consistency of the questionnaire. Removing any of the questions did not give any higher values of the Cronbach’s alpha.

The significant correlation between PUQE-scores and QOL-scores, nutritional intake and weight-change using Kruskal-Wallis test (displayed in Table 3), demonstrates the construct validity of the PUQE questionnaire. The significant reduction of PUQE-scores from admission to discharge (Table 4) likewise demonstrates the responsiveness of this questionnaire. Finally as displayed in S2 Table, there is no ceiling effect (defined as <15% of respondents reporting the highest PUQE-score = 15, [21]) while for the control group a certain floor effect was noted as 19% of patients reported the lowest PUQE-score = 3.

## Discussion

In this study we have validated SUKK, the Norwegian version of PUQE-24 in a Norwegian population. PUQE-scores were significantly higher in patients with severe HG compared to the control group. The food intake in patients with severe NVP/HG was found significantly lower than in the control group and the PUQE-scores were inversely correlated to the women’s food intake.

To our knowledge the direct relation between PUQE-scores and women’s comprehensive nutritional intake has not yet been evaluated in any study. Likewise the changes in PUQE-scores from admission to hospital discharge have not been described.

Our results are in line with the studies evaluating the English PUQE; whether evaluated during 12 hours [10], 24 hours [8] or as a mean during first trimester [9]; high scores are associated with severe NVP/HG and high PUQE-scores correlate with poor QOL.

Several studies have demonstrated the PUQE-score changes during outpatient antiemetic treatment [22,23]. Only one study has evaluated PUQE-scores during hospital treatment by evaluating a 5 days crossover RCT of clonidine versus placebo in 12 hospitalized HG patients [15]. In our study we directly compare scores of PUQE at admission with those at discharge, demonstrating that after hospital treatment PUQE-scores and QOL-scores were significantly improved.

Severe NVP/High PUQE-scores have been validated to associate with stop or significantly reduced intake of multivitamin supplementation, a surrogate marker of insufficient nutritional intake [8–10]. In the study from Montreal [9] 24 hours fluid intake did not correlate with PUQE-scores but with reduced well-being. In our study, a high PUQE-score is consistent with a woman being at serious nutritional risk. The different weight changes demonstrated (weight loss in the high PUQE-score groups compared to increased weight in the lowest score group) strengthen the PUQE-score as predictor of insufficient nutrition.

A self-selection bias caused by a higher interest in food and health in those willing to participate in a study, as compared to the general population [24,25], could have distorted the control group in favor of reduced frequency of nausea, this should have overestimated differences towards the hyperemesis group. However our control group may seem more similar to a NVP-group than women without any nausea. Still the median PUQE-score of 7 in our control group is comparable to the mean 6.7 of the routine prenatal cohort from Montreal [9] and their gestational length of 11.0 weeks was similar to our control group of 11.9 weeks. Also the control women in our study had gained weight as compared to the weight loss reported from the hospitalized HG patients. Overall we deem our control group as sufficient representative for Norwegian pregnant women.

The HG patients had a median gestational age of 65 days or 9.3 weeks, this is comparable to a group of 50 HG patients participating in a British Hyperemesis Impact of Symptoms Questionnaire (HIS) study with 9.8 weeks gestation [26]. Their control group also had somewhat longer gestational age at inclusion; 12.7 weeks as compared to 11.9 weeks in our control group. Including > 50% of eligible patients at the recruiting hospitals we do consider our HG group as representative. Although our patients and controls had different proportions regarding gestational length, both groups contained patients throughout the gestational period aimed for and enough so to effectively display the different effect of the PUQE-score for hyperemesis versus normal patients.

Although we included more than the estimated number of 28 participants in each group as indicated by the power calculation, our study of 38 HG patient is not large. However, the main studies validating the English PUQE had even smaller samples of HG n = 21 [10] or severe NVP defined as PUQE-score ≥13; n = 16 [8] and n = 9 [9]. Similarly when PUQE was evaluated in Spanish, this was tested by 10 women [8]. The South-African case-control study evaluating nutritional intake in HG included 20 women in each group [19]. Thus our study should be considered as sufficiently powered to evaluate these three aspects of PUQE-score in our cohort: discriminate severity of nausea, impact on quality of life and nutritional intake.

We aimed to compare the score of the PUQE form assessing the severity of NVP to the women’s food intake during 24 hours by using a self-reported food diary. The instruction was to fill in the list consecutively starting from the first morning at inclusion, as a real-time procedure, to minimize recall bias. The very good correlation between dietary intake and PUQE-scores gives us reason to believe that this information was reported at the same day. Since patients would start answering questionnaires the first morning after admission, the medication and fluid/nutritional regimen started at admission might already have alleviated some of their symptoms. This might lead to an underestimation of the differences between the groups.

The amount and the types of food that were consumed the day of registration can be affected by the fact that this is supposed to be registered. Women have a tendency to under-report what they eat [27]. The healthy controls might forget to report during busy every-day life. The hospitalized HG patients eating less and having fewer distractions may be more accurate in reporting, leading to an underestimation of differences between groups. A comprehensive food interview by nutritionist might have improved the nutritional registration. This is a time-consuming procedure that possibly would have hampered inclusion of control women as well as imposing an additional burden on the bed-ridden patients and therefor was waived.

The estimated nutritional intake in the control group (median 1652 kcal) is lower than the 2279 kcal reported by women without nausea in the Norwegian Mother and Child cohort [1]. However that study is based on women answering at 18–22 weeks a very comprehensive food frequency questionnaire (FFQ) encompassing their food habits as a mean of the first 4–5 months of pregnancy. This FFQ has been demonstrated to be representative compared to food diaries performed at 15–16 weeks of pregnancy and may be more representative for the women’s situation during 2^nd^ trimester rather than 1^st^ trimester. A Finnish cohort study of 187 women enrolled during 1^st^ trimester and using a 3-day food diary, estimated daily energy intake as 8339kJ/1993kcal for women without NVP and 8013kJ/1915kcal for those reporting any NVP [28]. Neither of these studies included HG patients. One South-African case-control study [19] found significantly lower energy intake for 20 HG patients (median 1035kcal) as compared to 20 healthy pregnant women (median 2374 kcal) using a dietary interview encompassing several days before inclusion/admission. The estimation of 990 kcal daily nutritional intake in our HG group is in line with this and thus considered valid. However the control group’s assessed intake of 1652 kcal/24 hours is probably an underestimation due to underreporting of food/drink intake. A caloric intake 28% less than recommended during first trimester is not considered compatible with their reported weight gain. Thus the nutritional differences between patients and controls are probably underestimated in our study. Still we do find that high PUQE-scores are significantly correlated with low nutritional intake.

Information regarding the women’s ethnicity was not collected. As the aim of study was to validate a questionnaire in Norwegian, the participants had to be well knowledgeable, preferable native speakers, of Norwegian. Thus, participants would be assumed to be mainly Norwegian or Scandinavian.

In assessing quality of health questionnaires several criteria have been proposed as important; content validity, criterion validity, reproducibility, internal consistency, construct validity, responsiveness, floor and ceiling effect, and interpretability [21]. The first three criteria are essential when constructing and validating a new questionnaire, comparing to a former “gold standard”. This has been done when PUQE was developed [7,10] and was not the scope of our study. But we have demonstrated a good internal consistency of the PUQE-score with a Cronbach’s alpha of 0.846 and good construction validity as the association between PUQE-scores and QOL-scores, nutritional intake and weigh-change were all highly significant. Responsiveness defined as ability to detect clinically important changes over time has been demonstrated as PUQE-scores from the HG patients were significantly reduced from admission to discharge. A reduction in score from median 13 to 6 corresponds to belonging in the severe NVP/HG category (range 13–15) at admission and leaving hospital as in the mild NVP category (range 3–6), this we will deem as highly clinical meaningful and thus representing good interpretability. No ceiling effect has been detected and only for the control group a certain floor effect is demonstrated. Actually a value of 3 means no nausea, vomiting or retching, thus it is not meaningful to construct an even lower category.

The strength of this study is that we have stringently translated and back-translated a questionnaire that has earlier been validated in several languages among different cohorts of pregnant women. Our study has been conducted prospectively and findings from former studies have been replicated in a Norwegian setting. In addition we have validated this questionnaire to identify hyperemesis patients being at severe nutritional risk. The PUQE questionnaire now can be considered a simple but valuable tool to identify women with severe NVP/HG in need of hospital treatment in a Scandinavian population. In research settings PUQE should be recommended for classifying the degree of NVP as well as evaluating the impact of QOL. However further studies are needed to demonstrate the performance of the questionnaire in a clinical setting; guiding and monitoring the effect of antiemetic and nutritional interventions.

## Conclusion

This prospective cohort validation study demonstrated that SUKK, the Norwegian translated version of PUQE-24, is valid as a clinical tool to distinguish between regular morning sickness and severe nausea and vomiting of pregnancy/HG. Additionally, a strong inverse correlation between the scores of the PUQE questionnaire and the self-reported food intake and weight gain at inclusion for the participating women was demonstrated. Furthermore, our study demonstrated that after hospital treatment the PUQE-score decreases, and the quality of life score, QOL, increases.

## Supporting Information

S1 Fig SUKK*-questionnaire; the Norwegian translation of PUQE-24^¤^-questionnaire used in prospective cohort validation study of Hyperemesis Gravidarum versus healthy pregnant women. *SvangerskapsUtløst Kvalme Kvantifisering, ^¤^Pregnancy-Unique Quantification of Emesis and nausea.
(TIF)

S2 Fig Quality of Life Score in HG^ patients at admission (n = 37), healthy pregnant women (n = 31) and HG^ patients at discharge (n = 35). ^Hyperemesis Gravidarum.
(TIF)

S3 Fig Nutritional intake (kcal/24h) is inversely correlated to PUQE-24*-scores in HG^ patients and healthy pregnant women. *Pregnancy-Unique Quantification of Emesis and nausea. ^Hyperemesis Gravidarum.
(TIFF)

S1 Table Nutritional form used for 24 hours prospectively registration in a Norwegian cohort validation study of Hyperemesis Gravidarum versus healthy pregnant women. (DOCX)

S2 Table Range of PUQE-24*-scores from all study participants; healthy pregnant women at inclusion and patients with HG^ at admission and discharge from hospital. *Pregnancy-Unique Quantification of Emesis and nausea, ^Hyperemesis Gravidarum.
(DOCX)

S3 Table PUQE-24*-scores from patients discharged after hospital treatment for HG^ compared to healthy pregnant women (controls). *Pregnancy-Unique Quantification of Emesis and nausea, ^Hyperemesis Gravidarum.
(DOCX)

S4 Table PUQE-24* categories compared to nutritional intake during 24 hours in cohort study of HG^ patients (n = 37) and healthy pregnant women (n = 31). *Pregnancy-Unique Quantification of Emesis and nausea, ^Hyperemesis Gravidarum.
(DOCX)

## References

[1] Chortatos A, Haugen M, Iversen P, Vikanes A, Magnus P, Veirod M. Nausea and vomiting in pregnancy: associations with maternal gestational diet and lifestyle factors in the Norwegian Mother and Child Cohort Study. BJOG. 2013;13: 1642–1653. 10.1111/1471-0528.12406 23962347

[2] Power Z, Thomson AM, Waterman H. Understanding the stigma of hyperemesis gravidarum: qualitative findings from an action research study. Birth. 2010;37: 237–244. 10.1111/j.1523-536X.2010.00411.x 20887540

[3] Verberg MF, Gillott DJ, Al-Fardan N, Grudzinskas JG. Hyperemesis gravidarum, a literature review. Hum Reprod Update. 2005;11: 527–539. 16006438 10.1093/humupd/dmi021

[4] Fairweather DV. Nausea and vomiting in pregnancy. Am J Obstet Gynecol 1968;102: 135–175. 4877794 10.1016/0002-9378(68)90445-6

[5] Vandraas K, Vikanes A, Vangen S, Magnus P, Stoer N, Grjibovski A. Hyperemesis gravidarum and birth outcomes-a population-based cohort study of 2.2 million births in the Norwegian Birth Registry. BJOG. 2013;13: 1654–1660. 10.1111/1471-0528.12429 24021026

[6] Dodds L, Fell DB, Joseph KS, Allen VM, Butler B. Outcomes of pregnancies complicated by hyperemesis gravidarum. Obstet Gynecol. 2006;107: 285–292. 16449113 10.1097/01.AOG.0000195060.22832.cd

[7] Koren G, Boskovic R, Hard M, Maltepe C, Navioz Y, Einarson A. Motherisk-PUQE (pregnancy-unique quantification of emesis and nausea) scoring system for nausea and vomiting of pregnancy. Am J Obstet Gynecol. 2002;186: S228–231. 12011891 10.1067/mob.2002.123054

[8] Ebrahimi N, Maltepe C, Bournissen FG, Koren G. Nausea and vomiting of pregnancy: using the 24-hour Pregnancy-Unique Quantification of Emesis (PUQE-24) scale. J Obstet Gynaecol Can. 2009;31: 803–807. 19941704 10.1016/S1701-2163(16)34298-0

[9] Lacasse A, Rey E, Ferreira E, Morin C, Berard A. Validity of a modified Pregnancy-Unique Quantification of Emesis and Nausea (PUQE) scoring index to assess severity of nausea and vomiting of pregnancy. Am J Obstet Gynecol. 2008;198: 71 e71–77. 10.1016/j.ajog.2007.05.051 18166311

[10] Koren G, Piwko C, Ahn E, Boskovic R, Maltepe C, Einarson A et al Validation studies of the Pregnancy Unique-Quantification of Emesis (PUQE) scores. J Obstet Gynaecol. 2005;25: 241–244. 16147725 10.1080/01443610500060651

[11] Lombardi DG, Istwan NB, Rhea DJ, O'Brien JM, Barton JR. Measuring outpatient outcomes of emesis and nausea management in pregnant women. Manag Care. 2004;13: 48–52. 15595402

[12] Maltepe C, Koren G. The management of nausea and vomiting of pregnancy and hyperemesis gravidarum-a 2013 update. J Popul Ther Clin Pharmacol. 2013;20: e184–192. 23863612

[13] Wibowo N, Purwosunu Y, Sekizawa A, Farina A, Tambunan V, Bardosono S. Vitamin B(6) supplementation in pregnant women with nausea and vomiting. Int J Gynaecol Obstet. 2012;116: 206–210. 10.1016/j.ijgo.2011.09.030 22189065

[14] Albayrak M, Karatas A, Demiraran Y, Erman H, Topuz S, Biyik I et al Ghrelin, acylated ghrelin, leptin and PYY-3 levels in hyperemesis gravidarum. J Matern Fetal Neonatal Med. 2013;26: 866–870. 10.3109/14767058.2013.766699 23330872

[15] Maina A, Arrotta M, Cicogna L, Donvito V, Mischinelli M, Todros T et al Transdermal clonidine in the treatment of severe hyperemesis. A pilot randomised control trial: CLONEMESI. BJOG. 2014;12: 1556–1562. 10.1111/1471-0528.12757 24684734

[16] ClinicalTrials.gov. Pregnancy Specific Nausea Questionnaire (PUQE) Translated and Tested in Norwegian (PUQE-N). Available: http://clinicaltrials.gov/ct2/show/NCT01836835. Accessed 05 February 2015.

[17] Guttormsen A, Hensrud A, Irtun Ø, Mowé M, Sørbye LW, Thoresen L et. al Nasjonale faglige retningslinjer for forebyggende og behandling av underernæring. In: Ernæring HA editor. Oslo: Helsedirektoratet; 2009.

[18] Helsedirektoratet. Anbefalinger om kosthold, ernæring og fysisk aktivitet. Oslo: Helsedirektoratet; 2014.

[19] van Stuijvenberg ME, Schabort I, Labadarios D, Nel JT. The nutritional status and treatment of patients with hyperemesis gravidarum. Am J Obstet Gynecol. 1995;172: 1585–1591. 7755076 10.1016/0002-9378(95)90501-4

[20] von Elm E, Altman DG, Egger M, Pocock SJ, Gotzsche PC, Vandenbroucke JP et al The Strengthening the Reporting of Observational Studies in Epidemiology (STROBE) statement: guidelines for reporting observational studies. J Clin Epidemiol. 2008;61: 344–349. 10.1016/j.jclinepi.2007.11.008 18313558

[21] Terwee CB, Bot SD, de Boer MR, van der Windt DA, Knol DL, Dekker J et al Quality criteria were proposed for measurement properties of health status questionnaires. J Clin Epidemiol. 2007;60: 34–42. 17161752 10.1016/j.jclinepi.2006.03.012

[22] Klauser CK, Fox NS, Istwan N, Rhea D, Rebarber A, Desch C et al Treatment of severe nausea and vomiting of pregnancy with subcutaneous medications. Am J Perinatol. 2011;28: 715–721. 10.1055/s-0031-1280594 21667429

[23] Boskovic R, Einarson A, Maltepe C, Wolpin J, Koren G. Diclectin therapy for nausea and vomiting of pregnancy: effects of optimal dosing. J Obstet Gynaecol Can. 2003;25: 830–833. 14532951 10.1016/s1701-2163(16)30673-9

[24] Meltzer HM, Brantsaeter AL, Ydersbond TA, Alexander J, Haugen M. Methodological challenges when monitoring the diet of pregnant women in a large study: experiences from the Norwegian Mother and Child Cohort Study (MoBa). Matern Child Nutr. 2008;4: 14–27. 10.1111/j.1740-8709.2007.00104.x 18171404 PMC6860710

[25] Gibson R. Principles of Nutritional Assessment. New York, USA: Oxford University Press; 2005.

[26] Power Z, Campbell M, Kilcoyne P, Kitchener H, Waterman H. The Hyperemesis Impact of Symptoms Questionnaire: development and validation of a clinical tool. Int J Nurs Stud. 2010;47: 67–77. 10.1016/j.ijnurstu.2009.06.012 19646694

[27] Stubbs RJ, O'Reilly LM, Whybrow S, Fuller Z, Johnstone AM, Livingstone MB et al Measuring the difference between actual and reported food intakes in the context of energy balance under laboratory conditions. Br J Nutr. 2014; 1–12. 10.1017/S0007114514000154 24635904

[28] Latva-Pukkila U, Isolauri E, Laitinen K. Dietary and clinical impacts of nausea and vomiting during pregnancy. J Hum Nutr Diet. 2010;23: 69–77. 10.1111/j.1365-277X.2009.01019.x 19943842

