# Supplementary figures and images for: Norwegian PUQE (Pregnancy-Unique Quantification of Emesis and Nausea) Identifies Patients with Hyperemesis Gravidarum and Poor Nutritional Intake: A Prospective Cohort Validation Study
Source: PLoS One. 2015 Apr 1;10(4):e0119962. doi: 10.1371/journal.pone.0119962 (PMC4382206; doi:10.1371/journal.pone.0119962)

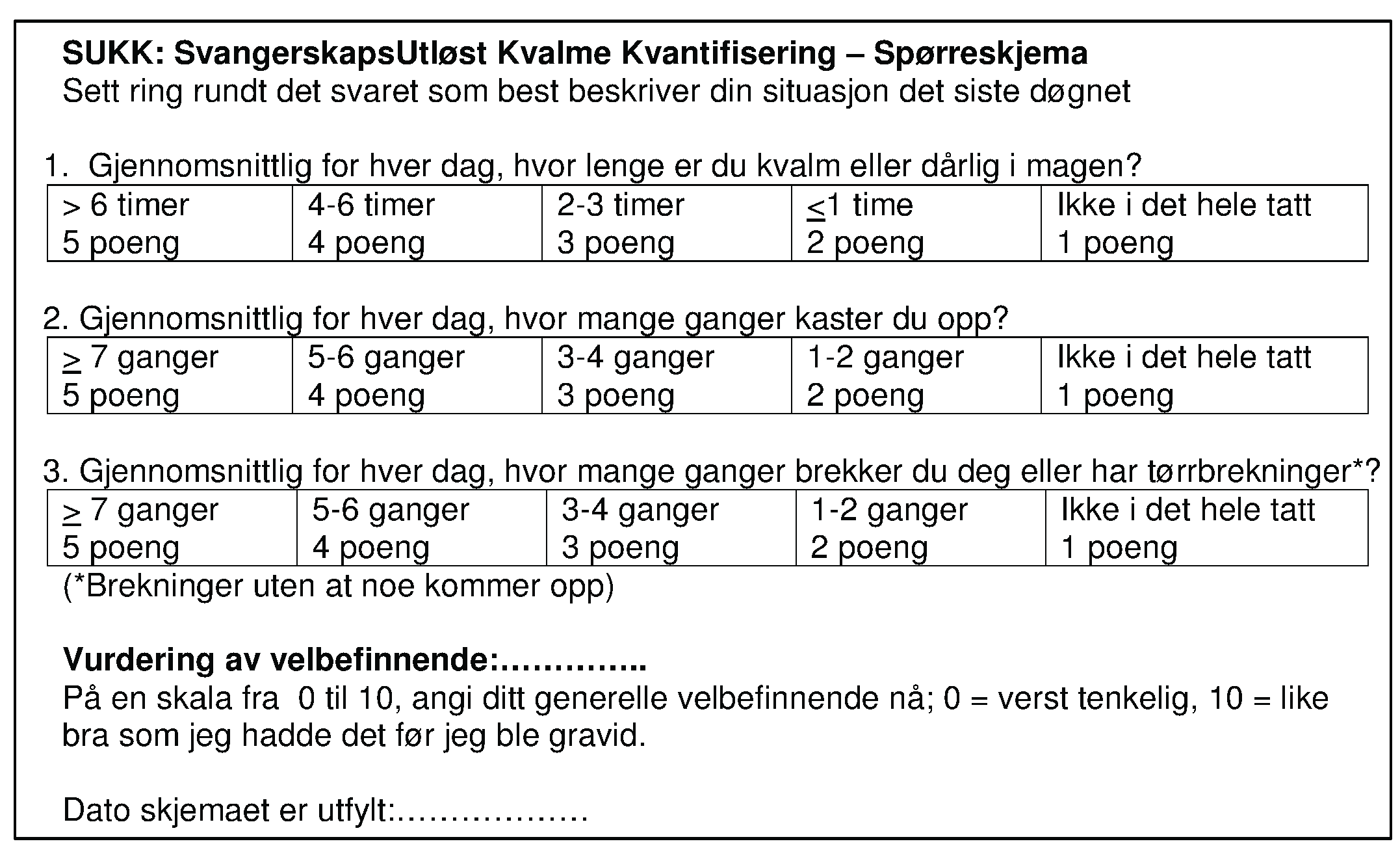

Supplement: S1 Fig — *SvangerskapsUtløst Kvalme Kvantifisering, ¤Pregnancy-Unique Quantification of Emesis and nausea. (TIF) [file pone.0119962.s001.tif]

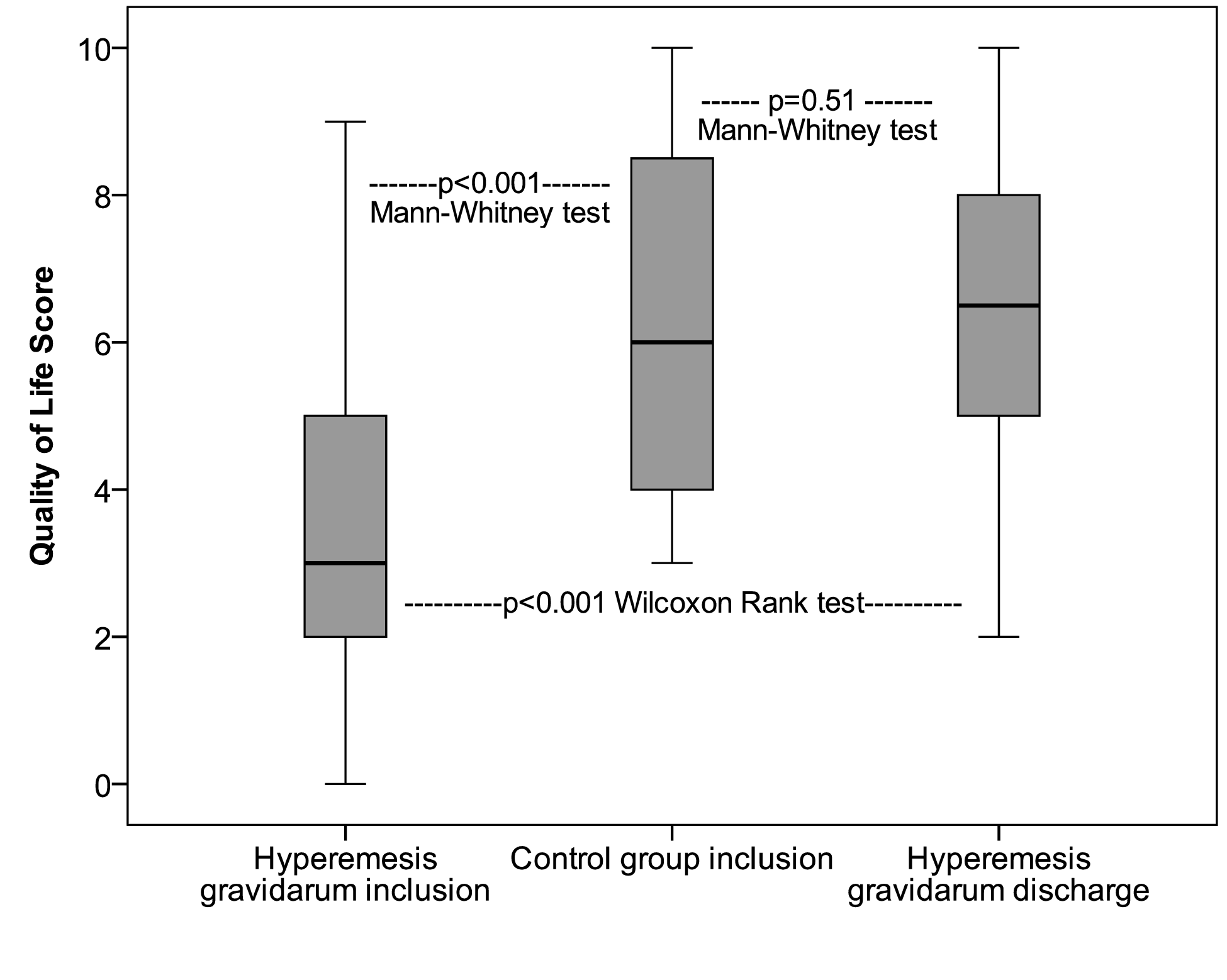

Supplement: S2 Fig — ^Hyperemesis Gravidarum. (TIF) [file pone.0119962.s002.tif]

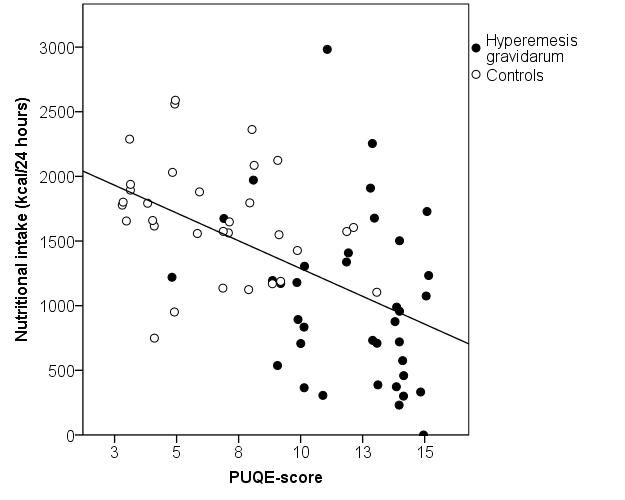

Supplement: S3 Fig — *Pregnancy-Unique Quantification of Emesis and nausea. ^Hyperemesis Gravidarum. (TIFF) [file pone.0119962.s003.tiff]
