# Supplementary material for: Norwegian PUQE (Pregnancy-Unique Quantification of Emesis and Nausea) Identifies Patients with Hyperemesis Gravidarum and Poor Nutritional Intake: A Prospective Cohort Validation Study
Source: PLoS One. 2015 Apr 1;10(4):e0119962. doi: 10.1371/journal.pone.0119962 (PMC4382206; doi:10.1371/journal.pone.0119962)
Supplement: S1 Table — (DOCX) [file pone.0119962.s004.docx]

**Table S1. Nutritional form used for 24 hours prospectively registration in a Norwegian cohort validation study of Hyperemesis Gravidarum (HG) versus healthy pregnant women.**

| SUKK-S Egenrapportert matinntaksskjema | | |
| --- | --- | --- |
| **MATVARE** | **ENHET** | **ANTALL/MENGDE SPIST** |
| Kneipp/grovbrød | 1/2 skive* |  |
| Loff | 1/2 skive* |  |
| Rundstykke | 1/2 skive* |  |
| Knekkebrød | 1 stk* |  |
| Frokostblanding | 1 porsj u/melk |  |
| Corn flakes | 1 porsj u/melk |  |
| Havregrøt | 1 porsjon |  |
| Risgrøt | 1 posjon |  |
| Egg | 1 stk |  |
| Yoghurt | 1 beger |  |
| Youghurt(duokartong) | 1 beger |  |
| Is | 1 beger |  |
| Eple/Appelsin | 1 stk |  |
| Banan | 1 stk |  |
| 10 druer | 1 porsjon |  |
| Midag | 1 porsjon |  |
| Dessert | 1 porsjon |  |
| Suppe(salt) | 1 porsjon |  |
| Havresuppe(melk) | 1 porsjon |  |
| Havresuppe(vann) | 1 porsjon |  |
| Kake/vaffelplate | 1 stk |  |
| Tørr kjeks | 1 stk |  |
| Bolle | 1 stk |  |
| *Evt annen mat:* |  |  |
| H-melk, kefir | 1 glass/1,5 dl |  |
| Lettmelk/Biola | 1 glass/1,5 dl |  |
| Skummet melk(søt/sur) | 1 glass/1,5 dl |  |
| Appelsinjuice | 1 glass/1,5 dl |  |
| Saft/Brus | 1 glass/1,5 dl |  |
| Vann/Farris/sukkefri brus | 1 glass/1,5 dl |  |
| Kaffe/Te u sukker | 1 glass/1,5 dl |  |
| Vin | 1 glass/1,5 dl |  |
| Øl | 1 glass/1,5 dl |  |
| Næringsdrikk | 1 boks |  |
| *Evt. annen drikke:* |  |  |
| Sukkerbit | 1 stk |  |
| Karameller/drops | 1 stk |  |
| Sjokolade (60g) | 1 stk |  |
| Peanutter | 15g/ca 20stk |  |
| Potetgull | 15g/1dl |  |
| *Evt. annet "ekstra":* |  |  |
| Skjemaet fylles ut for ett døgn. Marker etter hvert som du spiser og drikker | | |
| med å krysse av for den enkelte matenhet (X evt I). | | |
| Spiser du mindre enn en enhet anføres det, f.eks 1/2 glass skriv 1/2 | | |
| *Inkludert pålegg  SUKK:SvangerskapsUtløstKvalmeKvantifisering |  |  |
