# Supplementary material for: Norwegian PUQE (Pregnancy-Unique Quantification of Emesis and Nausea) Identifies Patients with Hyperemesis Gravidarum and Poor Nutritional Intake: A Prospective Cohort Validation Study
Source: PLoS One. 2015 Apr 1;10(4):e0119962. doi: 10.1371/journal.pone.0119962 (PMC4382206; doi:10.1371/journal.pone.0119962)
Supplement: S2 Table — *Pregnancy-Unique Quantification of Emesis and nausea, ^Hyperemesis Gravidarum. (DOCX) [file pone.0119962.s005.docx]

**Table S2. Range of PUQE-24*-scores from all study participants; healthy pregnant women at inclusion and patients with HG^ at admission and discharge from hospital**.

|  | All participants at inclusion | | Patients inclusion | | Controls inclusion | | Patients discharge |  |
| --- | --- | --- | --- | --- | --- | --- | --- | --- |
| PUQE-score | number | % | number | % | number | % | number | % |
| 3 | 6 | 6 | 0 | 0 | 6 | 19 | 0 | 0 |
| 4 | 4 | 4 | 0 | 0 | 4 | 13 | 10 | 27 |
| 5 | 5 | 5 | 1 | 3 | 4 | 13 | 5 | 14 |
| 6 | 2 | 2 | 0 | 0 | 2 | 6 | 5 | 14 |
| 7 | 5 | 5 | 1 | 3 | 4 | 13 | 3 | 8 |
| 8 | 5 | 5 | 1 | 3 | 4 | 13 | 4 | 11 |
| 9 | 7 | 7 | 3 | 8 | 4 | 13 | 5 | 14 |
| 10 | 7 | 7 | 6 | 16 | 1 | 3 | 1 | 3 |
| 11 | 2 | 2 | 1 | 5 | 0 | 0 | 2 | 5 |
| 12 | 4 | 4 | 1 | 5 | 2 | 6 | 0 | 0 |
| 13 | 7 | 7 | 6 | 16 | 1 | 3 | 0 | 0 |
| 14 | 10 | 9 | 10 | 27 | 0 | 0 | 1 | 3 |
| 15 | 5 | 5 | 5 | 14 | 0 | 0 | 0 | 0 |

*PUQE: Pregnancy-Unique Quantification of Emesis and nausea, ^Hyperemesis Gravidarum
