# Supplementary material for: Norwegian PUQE (Pregnancy-Unique Quantification of Emesis and Nausea) Identifies Patients with Hyperemesis Gravidarum and Poor Nutritional Intake: A Prospective Cohort Validation Study
Source: PLoS One. 2015 Apr 1;10(4):e0119962. doi: 10.1371/journal.pone.0119962 (PMC4382206; doi:10.1371/journal.pone.0119962)
Supplement: S3 Table — *Pregnancy-Unique Quantification of Emesis and nausea, ^Hyperemesis Gravidarum. (DOCX) [file pone.0119962.s006.docx]

**Table S3. PUQE-24*-scores from patients discharged after hospital treatment for HG compared to healthy pregnant women (controls).**

| Variable | Controls  n=31  Median 95% CI | | HG discharge  n=37  Median 95% CI | | *p*-value  Mann-Whitney  U-test |
| --- | --- | --- | --- | --- | --- |
| Question 1 score (length of nausea) | 3 | 2-4 | 3 | 2-4 | 0.714 |
| Question 2 score (rate of vomiting) | 1 | 1-1 | 1 | 1-1 | 0.873 |
| Question 3 score (rate of retching) | 2 | 2-2 | 2 | 1-2 | 0.456 |
| PUQE-score^a^ | 7 | 5-8 | 6 | 5-8 | 0.833 |
| Quality of life (QOL) score ^b^ | 6 | 4.5-8 | 7 | 6-8 | 0.509 |
|  |  |  |  |  |  |
| PUQE-score severity | Number | % | Number | % | *p*-value  Chi-square |
| Mild NVP^c^ (score <7) | 15 | 48 | 20 | 54 | 0.896 |
| Moderate NVP (score 7-12) | 15 | 48 | 16 | 43 |  |
| Severe NVP/HG (score 13-15) | 1 | 3 | 1 | 3 |  |

*Pregnancy Unique Quantification of Emesis and nausea, ^Hyperemesis Gravidarum ^a^Sum of Question 1, 2 and 3, ^b^Data from two patients at discharge are missing, ^c^NVP: Nausea and vomiting of pregnancy
