# Supplementary material for: Norwegian PUQE (Pregnancy-Unique Quantification of Emesis and Nausea) Identifies Patients with Hyperemesis Gravidarum and Poor Nutritional Intake: A Prospective Cohort Validation Study
Source: PLoS One. 2015 Apr 1;10(4):e0119962. doi: 10.1371/journal.pone.0119962 (PMC4382206; doi:10.1371/journal.pone.0119962)
Supplement: S4 Table — *Pregnancy-Unique Quantification of Emesis and nausea, ^Hyperemesis Gravidarum. (DOCX) [file pone.0119962.s007.docx]

**Table S4. PUQE-24* categories compared to nutritional intake during 24 hours in cohort study of HG^ patients (n=37) and healthy pregnant women (n=31).**

| Variable | Mild NVP^b^  PUQE-score <7  n=16  Median 95% CI | | Moderate NVP  PUQE-score 7-12  n=29  Median 95% CI | | Severe NVP/HG  PUQE-score 13-15  n=23  Median 95% CI | | *p*-value  Kruskal-  Wallis test |
| --- | --- | --- | --- | --- | --- | --- | --- |
| Energy intake (kcal^c^) | 1796 | 1558-2031 | 1408 | 1171-1605 | 878 | 459-1233 | <0.001 |
| Protein (g) | 69 | 48-80 | 48 | 40-57 | 26 | 11-44 | <0.001 |
| Fat (g) | 67 | 45-89 | 48 | 42-68 | 29 | 19-47 | 0.001 |
| Carbohydrates (g) | 213 | 156-251 | 167 | 149-200 | 100 | 59-169 | 0.004 |
| Vitamin D (µg) | 2 | 1-5 | 2 | 1-2 | 1 | 0-1 | <0.001 |
| Vitamin C (mg) | 104 | 67-162 | 75 | 53-132 | 49 | 15-65 | <0.001 |
| Vitamin B12 (µg) | 3 | 2-4 | 2 | 1-2 | 1 | 0-1 | <0.001 |
| Calcium (mg) | 701 | 454-896 | 491 | 329-673 | 228 | 180-397 | <0.001 |
| Iron (mg) | 9 | 5-10 | 5 | 4-6 | 3 | 1-4 | <0.001 |
| Magnesium (mg) | 277 | 204-332 | 209 | 143-242 | 110 | 58-171 | <0.001 |
| Sodium (mg) | 2059 | 1665-2489 | 1729 | 1316-2036 | 1267 | 722-1532 | <0.001 |
| Fiber (g) | 20 | 13-28 | 13 | 10-18 | 8 | 5-10 | <0.001 |

*PUQE: Pregnancy-Unique Quantification of Emesis and nausea, ^Hyperemesis Gravidarum ^a^QOL: Quality of Life, ^b^NVP: Nausea and vomiting of pregnancy, ^c^kcal: kilocalories
